# Nanomotion Detection-Based Rapid Antibiotic Susceptibility Testing

**DOI:** 10.3390/antibiotics10030287

**Published:** 2021-03-10

**Authors:** Sandor Kasas, Anton Malovichko, Maria Ines Villalba, María Elena Vela, Osvaldo Yantorno, Ronnie G. Willaert

**Affiliations:** 1 Laboratory of Biological Electron Microscopy, Ecole Polytechnique Fédérale de Lausanne, 1015 Lausanne, Switzerland; anton.malovichko@epfl.ch (A.M.); villalaine@gmail.com (M.I.V.); 2 Unité Facultaire d’Anatomie et de Morphologie (UFAM), CUMRL, University of Lausanne, 1005 Lausanne, Switzerland; 3 International Joint Research Group VUB-EPFL NanoBiotechnology and NanoMedicine (NANO), Vrije Universiteit Brussel, 1050 Brussels, Belgium; Ronnie.Willaert@vub.be; 4 Instituto de Investigaciones Fisicoquímicas Teóricas y Aplicadas (INIFTA), Facultad de Ciencias Exactas, Universidad Nacional de La Plata, and CONICET, Diagonal 113 y 64, 1900 La Plata, Argentina; mevela@inifta.unlp.edu.ar; 5 Centro de Investigación y Desarrollo en Fermentaciones Industriales (CINDEFI-CONICET-CCT La Plata), Facultad de Ciencias Exactas, Universidad Nacional de La Plata, 1900 La Plata, Argentina; yantorno@quimica.unlp.edu.ar; 6 Research Group Structural Biology Brussels, Vrije Universiteit Brussel, 1050 Brussels, Belgium

**Keywords:** rapid antibiotic susceptibility testing (AST), nanomotion, atomic force microscopy (AFM), *B. pertussis*

## Abstract

Rapid antibiotic susceptibility testing (AST) could play a major role in fighting multidrug-resistant bacteria. Recently, it was discovered that all living organisms oscillate in the range of nanometers and that these oscillations, referred to as nanomotion, stop as soon the organism dies. This finding led to the development of rapid AST techniques based on the monitoring of these oscillations upon exposure to antibiotics. In this review, we explain the working principle of this novel technique, compare the method with current ASTs, explore its application and give some advice about its implementation. As an illustrative example, we present the application of the technique to the slowly growing and pathogenic *Bordetella pertussis* bacteria.

## 1. Introduction

According to a WHO report [1], a post-antibiotic era—in which common infections and minor injuries can kill—is far from being an apocalyptic fantasy but a very real possibility for our century. This is due to the fast emergence of multidrug-resistant microbial pathogens, which is caused by the extensive, sometimes unnecessary use of antimicrobials and the lack of interest of pharma in developing new compounds. The cost of antimicrobial resistance (AMR) is projected to increase significantly as some models predict a rise in global casualties from the present figure of one million to 10 million in 2050 [2]. To combat the rise of AMR, a profound understanding of the mechanisms of microbial infections, the development of new diagnostic tools and new antimicrobials are necessary.

To rationalize the use of large spectrum antimicrobial drugs, it is essential to have a rapid and sensitive detection system that identifies the most appropriate drug to fight a given microorganism immediately at the admission of the patient in a medical center. Current antimicrobial susceptibility testing (AST) technologies mostly rely on microbial culturing and thus replication, which can therefore take up to 1 to 3 days [3,4]. As a result of the diagnostic’s limited speed, accurate treatment, with effective narrow-range antimicrobial agents, is often replaced by the use of broad-spectrum antimicrobials [5,6,7]. The overuse of broad-spectrum antibiotics accelerates the further rise of AMR worldwide [5]. The development of rapid AST technologies is thus important in the battle against AMR. Rapid AST technologies can therefore have a double effect, firstly increasing the survival rate for patients with infections, and secondly, it could potentially extend the lifespan of current narrow-spectrum antimicrobials [8].

## 2. Current Antimicrobial Susceptibility Testing (AST) Methods

Fighting the threat of multidrug-resistant pathogens requires a multi-disciplinary approach in which rapid AST plays a critical role. The classical method to determine antibiotic susceptibility is the disk diffusion method [3,9,10,11]. This well-established method requires a growth period before the actual disk test is performed, which also is based on further growth during 16 to 20 h. Since some pathogenic bacteria are non-culturable, other methods have to be used. Therefore, new methods that also allow one to perform AST on non-culturable microorganisms in a short time frame [8] are needed. Current AST methods can be divided into phenotypic and molecular tests [12,13,14].

Phenotypic assays monitor the growth of the microorganism in the presence of antibiotics [15]. Classical AST methods are culture-based (Table 1). Since these methods mostly rely on microbial culturing and thus replication, the performance of these tests takes 1 to 3 days [3,4]. Agar dilution assays, i.e., disk diffusion and E-test methods, are flexible and simple methods that are commonly used in clinical microbiology labs (Table 1). They allow one to determine the minimal inhibitory concentration (MIC). A MIC test can also be used using broth dilution assays, where the MIC corresponds to the lowest concentration of antibiotic that completely inhibits bacterial growth and lacks visible turbidity [16]. Broth macrodilution assays have been miniaturized and automated [3]. Several commercial semi-automated or fully automated instruments have been developed, such as the MicroScan WalkAway, Vitek-2, BD Phoenix, Wider System and Sensititre system [3,4,7,17,18,19,20,21,22,23,24,25,26,27]. The time–kill test is a tool for obtaining information on the dynamic interaction between the antimicrobial and the microbial strain [14]. The time–kill curve reveals a time- or concentration-dependent antimicrobial effect and can be used to determine synergism or antagonism between drugs in combinations [28,29,30,31,32]. Optical-based AST methods have been developed to measure the growth rate, such as the “multiplexed automated digital microscopy (MADM)” method [33,34,35] and the oCelloscope [36], as well as to measure morphological changes of single cells upon antibiotic treatment [37] (Table 1). Recently, electrical-based AST methods that are based on impedance, capacitance, resistance and electrochemical measurements, and mechanical-based methods have also been developed (see Table 1 for some examples).

Molecular techniques rely on the determination of a particular fingerprint associated with the resistance to a specific antibiotic [15,48,49] (Table 2). Real-time PCR techniques and specifically constructed DNA microarrays have been developed to detect a spectrum of genes that could be related to resistance to different antibiotics [15,48,50]. Some of these techniques (e.g., the Xpert MTB/RIF assay [51,52,53]) have been commercialized and are characterized by a very high reliability and speed of execution [13]. In the last 10 years, various methods have been developed that are based on matrix-assisted laser desorption ionization time-of-flight mass spectrometry (MALDI-TOF MS) [54]. MALDI-TOF MS allows for the fast identification of the microbial species [55,56,57,58,59,60,61,62,63,64]. The use of MALDI-TOF MS for AST lies in the combination of MALDI TOF MS identification with an established AST method, such as the combination with Vitek-2 [65] or the BD Phoenix system [66,67]. MALDI-TOF MS has also been combined with stable isotope labeling by amino acid in cell culture (SILAC). This MS method can identify the metabolically inactive microorganisms due to the action of the antibiotic [68]. ATP bioluminence assays can provide a fast antibacterial [69,70,71], antimycobacterial [72,73] and antifungal testing [74,75] where the growth is determined based on the ATP quantification. Another molecular marker for growth that has been used is 16S rRNA [76].

In this review, we will essentially focus on a novel way to characterize the susceptibility of microorganisms to antibiotics. The technique relies on the detection of the nanometric scale oscillations that characterize living cells. Several years ago, our team demonstrated that all living organisms oscillate at a nanometric scale and that these oscillations end as soon the organism dies [77]. Highlighting such minute movements on a single microorganism requires highly sensitive devices such as atomic force microscopes (AFMs). These instruments are particularly adapted to such challenges, since they can detect displacements in the range of 0.1 Å with a temporal resolution in the range of microseconds. As an illustration, the typical distance between two carbon atoms in an organic molecule is about 2 Å. The very first and straightforward application of such a life monitor is rapid AST. The aim of this article is to describe the working principle of these novel devices, to review their contributions to the field of AST and to discuss their future applications.

## 3. The Atomic Force Microscope (AFM) and the Cantilever as a Mass Sensor

**Figure 1:**
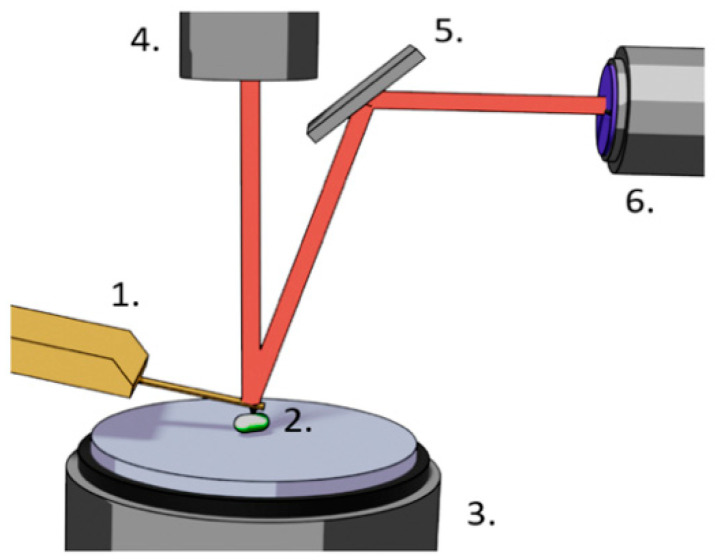
Components of a typical atomic force microscope (AFM). 1. Cantilever holding chip, cantilever and tip, 2. sample, 3. piezo electric sample holding stage, 4. laser, 5. mirror, 6. two- or four-segment photodiode.

The atomic force microscope was developed in late 1980s by Binning Quate and Gerber [78]. The instrument aimed to image non-conductive samples with atomic resolution. The microscope consists of a very sharp tip fixed at the end of a soft cantilever that scans the surface of the sample. The tip is maintained in contact with the surface and the deformations of the cantilever are used to reconstruct on a computer screen the 3D topography of the sample. The cantilever deformation is measured by monitoring the reflection angle of a laser beam that bounces off the end of the lever and ends its path on a two- or four-segment photodiode as depicted in Figure 1.

The sample, or in some cases the cantilever, are moved by piezo-electric crystals with a sub-Å precision [79]. These crystals convert an electric field that is applied on their surface into a mechanical strain that moves the sample. As an illustration, a potential difference of 100 V induces a displacement of 25 Å. The device can indifferently operate in vacuum air or liquid environments. This last ability makes the instrument highly interesting for biological applications. In the early days, the instrument was essentially used to image single molecules or individual cells in their “almost native” environment and very soon numerous microbiological applications of this novel imaging mode appeared in the literature. They essentially focused on the morphological alterations of bacteria following antibiotic exposure [80,81,82,83,84], or yeast cells upon antifungal treatment [85,86]. In the early days, the instrument was essentially used to image single molecules or individual cells in their “almost native” environment.

However, very soon it appeared that the device can also measure interaction forces between single molecules, monitor the mechanical properties of the sample at a nanometric resolution or measure minute changes in the mass of the samples attached to the cantilever. A comprehensive review of the different application domains of the AFM can be found in Krieg et al. [87]. The interaction force measurement is achieved by attaching a “ligand” molecule on the substrate and a “receptor” on the tip. To achieve the measurement, the cantilever is brought in close vicinity to the substrate to permit the attachment of the two molecular species. Eventually, the lever is moved afar the surface and sets the newly formed bond under stress which induces a downward bending of the cantilever. When the retraction force of the cantilever overcomes the adhesion between the two molecules, the bond breaks and the cantilever returns to its rest position. The maximal downward bending of the cantilever is directly proportional to the interaction force between the two molecules. The technique can also be used to explore intercellular interaction forces, by attaching mammalian, fungal or bacterial cells onto the lever and/or the substrate [88]. Since bacteria adhesive properties play an important role in biofilm formation, several teams have used AFM to explore this parameter on various surfaces [89,90,91]. The very same technique can be used to monitor the presence of specific molecules on bacterial surfaces [92,93,94].

The mechanical properties measurement of the sample by AFM consists of indenting (i.e., pushing) the tip into the sample and monitoring the deflection of the lever during the process. The harder the sample, the more the lever deforms. The curve that depicts the deformation of the cantilever as a function of the z position of the piezo electric crystal is referred to as a force distance curve (Fdc). By subtracting the Fdc obtained on the sample from another Fdc obtained on a hard substrate obtains a new curve referred to as an indentation curve. This last curve basically indicates the force that is required to indent the tip to a certain depth in the sample. The indentation curve of course depends on numerous other factors such as the shape of the tip [95]. The calculation of the sample’s elastic properties, i.e., its Young’s modulus, is obtained by fitting the indentation curve with a function such as the one of Hertz [96], Sneddon [97], JKR (Johnson Kendall Roberts) or Tatara [98]. The theoretical foundations of indentation curves are described elsewhere [99,100].

The first measurements of the mechanical properties of microorganisms by AFM were conducted on the archaebacterium *Methanospirillum hungatei* [101]. This first study was very quickly followed by numerous others involving *Magnetospirillum gryphiswaldense* [102], *Haemophilus influenzae* [103], *Pseudomonas aeruginosa* [104], *Klebsiella pneumoniae* [105] and *Staphylococcus aureus* [106]. Thanks to its very high spatial resolution, the AFM can also highlight specific domains that possess different mechanical properties than the rest of the cell wall. Arnal et al. demonstrated this capability of the microscope on *Bordetella pertussis* [107]. Stiffness inhomogeneities can also be highlighted underneath the bacterial surface by using a peculiar method to process Fdc. This AFM imaging mode is referred to as stiffness tomography [108,109]. It was used to monitor at high resolution stiffness modifications in bacteria upon antibiotic exposure [110,111].

For recent and comprehensive reviews on the use of the AFM to measure mechanical properties of the sample, one can refer to Kasas et al. [112] and Garcia [113], for various contributions of AFM in the field of microbiology, we refer to Garcia [113] and Formosa-Dague et al. [114].

Another type of measurement can also be carried out by the AFM: it involves the monitoring of the cantilever bending upon minute temperature variations [115,116] or the attachment of a molecule onto the lever. The measurement is achieved by monitoring the cantilever resonant frequency or its static bending upon a ligand molecule attachment. The cantilever resonant frequency changes due to the added mass whereas its static bending is induced by a change in the surface stress occurring on one side of the lever [117,118]. These types of devices are extremely sensitive and are widely applied as biosensors [117,119,120,121,122,123,124,125,126]. They have been used for the detection of very small masses [118,122,127,128,129], for measuring the buoyant mass of microorganisms and for determining the growth rates of individual microbial cells [130]. They were also applied to detect antibiotic–mucopeptides interaction on cantilever arrays [131].

AFM cantilevers have also been used as nanosensors for living cell studies since they offer many advantages. They are highly sensitive, selective, label-free and provide real-time in situ detection capabilities [132]. Experiment involving single cells have been reported for *Escherichia coli* [130,133,134], *Bacillus subtilis* [130,134], *Enterococcus faecalis* [133], *Saccharomyces cerevisiae* cells [130,132,133,135], HeLa cells [136], mouse lymphoblasts [130], and human lung carcinoma and mouse lymphocytic leukemia cells [133,137], mouse and human T cells [133]. Cell growth detection has been demonstrated by monitoring resonance frequency changes with immobilized *S. cerevisiae* and fungal *Aspergillus niger* spores [138].

The measurement of the cantilever resonant frequency can be quite challenging since the quality factor of the lever dramatically drops in liquids, due to the viscous forces that dampen its oscillations. An elegant workaround of the low-quality factor problem was recently found by Etayash et al. [139]. It consists of designing a channel embedded in the cantilever and of injecting a buffer containing the cells of interest. The cantilever resonant frequency is then measured in air and permits one to monitor the number of organisms in the channel and to distinguish viable and non-viable cells. The drawbacks of the technique are essentially the complexity of fabrication of such levers and the care that has to be taken during the operation to keep the channel clog- and bubble-free.

## 4. Nanomotion Detection

**Figure 2:**
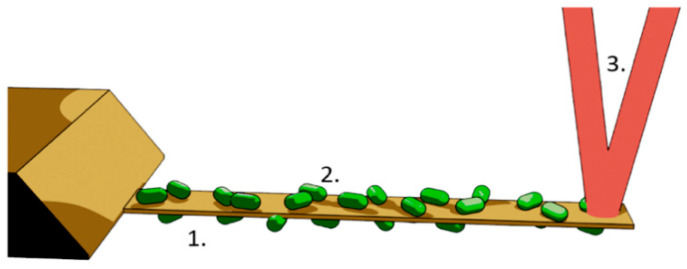
Setup to detect nanomotion of living organisms with an AFM cantilever. 1. AFM cantilever, 2. bacteria attached on both sides of the lever, 3. laser beam.

In early 2013, we noticed that living organisms deposited onto an AFM cantilever induce nanometric scale oscillations that immediately stop when the organism dies [77]. The setup as well as the measurement procedures are different from the previously mentioned mass and adhesion detection techniques. The organism of interest can be deposited on both sides of the lever as depicted in Figure 2 and the induced oscillations are far below the resonance frequency of the cantilever. The first experiments concerned motile bacteria (*Escherichia coli*); however, later studies revealed that “non-motile” microorganisms (i.e., those lacking propulsion mechanisms) induce such oscillations of the cantilever, too [140] (Table 3).

Subsequent extension of the study to larger cells such as yeast, plant and mammalian cells revealed that this phenomenon seems to be general for many organisms living on Earth (Table 4). Very quickly, we have foreseen the utility of such a detection method to conduct rapid antibiotic susceptibility tests and we therefore extended the technique to a large population of bacteria such as motile, non-motile, Gram-positive and Gram-negative germs as well to rapidly and slowly growing organisms. In all these experiments, the bacteria were exposed to different antibiotics and the organisms that were sensitive drastically reduced the cantilever oscillation amplitude in the minutes that followed the injection of the drug. Importantly, we also spotted the correlation that exists between the antibiotic concentration and the oscillation amplitude of the exposed microorganisms. This correlation permits one to draw dose–response curves and determine clinically important parameters such as the minimum inhibitory concentration (MIC) and minimum bactericidal concentration (MBC) [77]. Table 1 lists the biological samples that were explored with this method.

Importantly, other independent groups confirmed these results [142,143,148]. Interestingly, slowly growing bacteria such as *B. pertussis* or even mycobacteria also responded very quickly to the presence of antibiotics. These results emphasize further the potential of the technique as a rapid antibiotic susceptibility test, especially for tuberculosis and sepsis.

## 5. AFM Nanomotion Setup and Measurement

**Figure 3:**
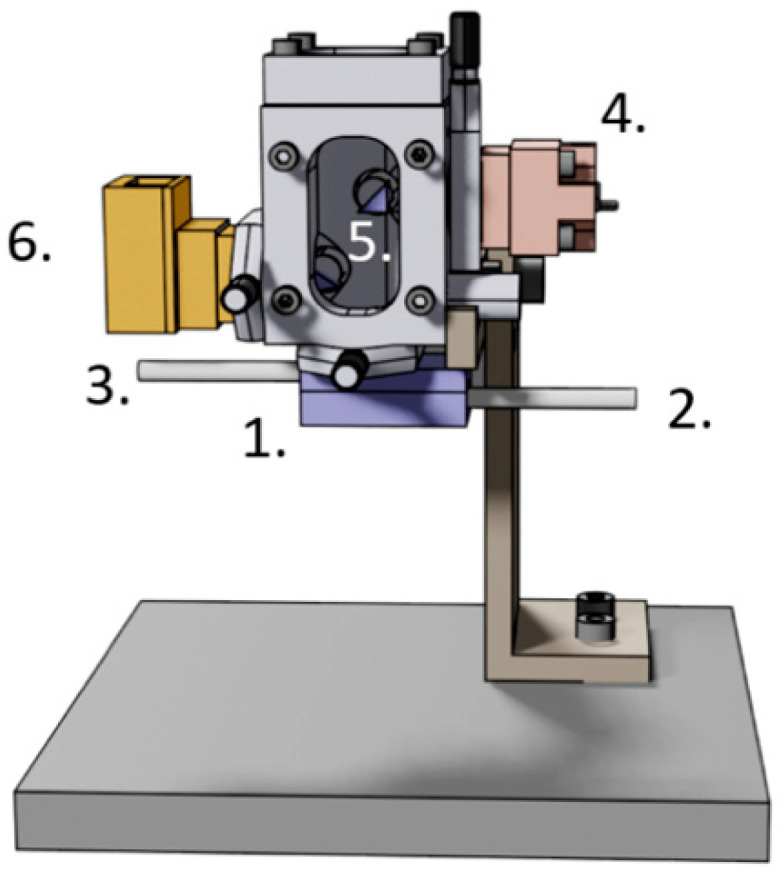
Dedicated nanomotion detection apparatus. 1. analysis chamber with the AFM cantilever, 2. inlet, 3. outlet, 4. laser inlet, 5. prisms to orient the laser beam onto the cantilever and the photodiodes, 6. photodiodes.

The technique is relatively simple to set up. A detailed procedure describing the preparation measurement and the data processing steps can be found in Venturelli et al. [150]. Briefly, the first step consists of functionalizing a relatively soft (0.06 N/m) AFM cantilever with a cross linking molecule such as glutaraldehyde, paraformaldehyde, APTES ((3-aminopropyl)triethoxysilane) or fibronectin. To ensure a stronger binding, we recommend a suspension of the microorganism in a phosphate-buffered saline (PBS) solution first. Cell membrane parts, various peptides or amino acids present in traditional culture media can hide the attachment spots on the cross-linking molecules. To ensure the attachment, the cantilever is immersed in a droplet containing the bacteria for about 15 min. The sensor is eventually inserted in the analysis chamber of the AFM to start the measurement. Biologically oriented instruments are preferable since they are designed to operate in liquids and permit one to exchange the “imaging” medium during the measurement. Custom built devices such the one depicted in Figure 3 can also be used.

The analysis chamber is then filled with the culture medium and the laser beam as well as the two- or four-segment photodiodes are adjusted to achieve the highest possible sensitivity. The measurement usually starts 5–10 min after the insertion of the cantilever into the analysis chamber. This delay permits the liquid medium and the cantilever to reach a thermal steady state. Typical measurements are carried out with a sampling rate of about 20 kHz. Such a high rate is preferred, since it permits one to capture the resonant frequency of the cantilever and to assess the correct position of the laser beam. The oscillations of the lever are recorded for about 15 min in the culture medium before the addition of the antibiotic. Usually, after 10–15 min exposure of bacteria to the drug, the oscillation amplitude drops if the bacteria are sensitive and remains stable or even increases if they are resistant. The experiment can be stopped at this stage; however, we usually inject an additional killing agent such as glutaraldehyde or paraformaldehyde to ensure that the oscillation amplitude drops to zero once all the organisms present on the cantilever are dead.

## 6. AFM Nanomotion Data Processing

The data processing step consists of a high pass filtering of the original data set to get rid of the thermal drift of the cantilever. The resulting data are eventually processed to extract the variance in a temporal window of 10 s. The variance of the signal is up to now the most sensitive parameter we found to distinguish between living and dead cells. A trial-and-error process in which we attempted to maximize the difference between signals recorded on living and dead samples determined the size of this 10 s window. It is important to mention that the amplitude of the variance signal directly correlates to the nutrient concentrations in the analysis chamber. This observation significantly extends the technique application domains.

Interestingly, frequency domain analysis did not reveal up to now any preferential peak (i.e., frequency) that we could attribute to the specific bacterial species or a metabolic state. However, we noticed that on the fast Fourier transforms (FFT) of the signal, the largest difference between living and dead cells is located between 0.2 and 100 Hz. This frequency window is very stable among all the living organisms that we, and other groups, explored up to now [142].

## 7. Application Example

**Figure 4:**
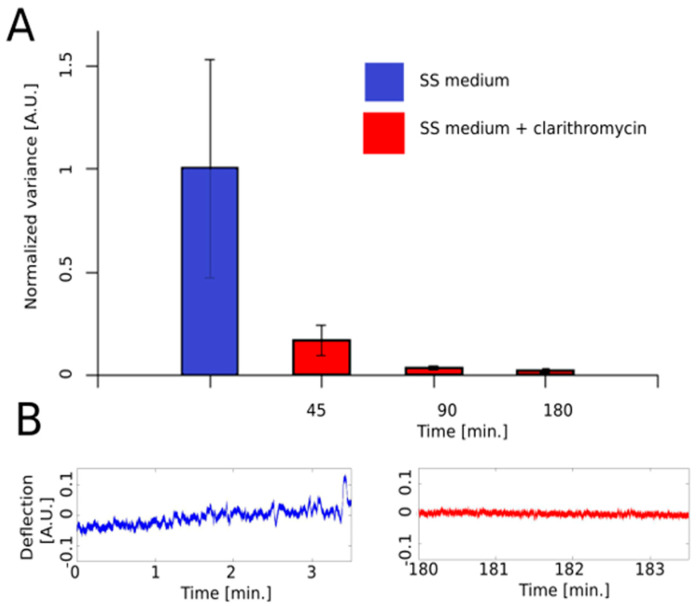
Illustration of the effect of clarithromycin on *B. pertussis* nanomotion. (**A**). Effect of the antibiotic at MIC on the variance of the nanomotion signal as a function of time before (blue column) and after (red columns) the exposure of the microorganism to the antibiotic. (**B**). Oscillations of the cantilever as a function of time before (blue curve) and after (red curve) the injection of the drug. SS medium: Stainer–Sholte liquid medium. The details of the preparation procedure are described in Appendix A.

To illustrate the technique and emphasize its susceptibility and rapidity, we present here an example of nanomotion-based AST applied to a slowly growing non-motile pathogenic bacterium. We selected *B. pertussis*, which is the causative agent of whooping cough. In this experiment, we monitored this by using one of our custom-made detectors for *B. pertussis* Tohama I strain nanomotion upon exposure to clarithromycin. The injection of the antibiotic at 5 µg/mL (i.e., MIC) in the analysis chamber induced within minutes a dramatic drop in the variance of the cantilever oscillations (Figure 4).

## 8. Advantages and Drawbacks of the AFM Nanomotion AST Technique.

Rapidity is probably the most straightforward advantage of the technique, especially in the case of tuberculosis or pertussis. A reliable AST obtained in about 1–2 h can be a “game changer” in the case of septicemia. The method does not require any previous knowledge about the microorganism to be tested and relies only on the bacterial phenotype. Finally, only a limited number of cells are required to conduct an AST. A few hundred bacteria adhered on the cantilever are enough to obtain a reliable and reproducible signal. This number can drop to one single cell in the case of larger microorganisms such as yeast. In the case of polymicrobial infections, a representative sample composed of the different bacterial species present in the patient has to be adhered to the cantilever. This will allow one to evaluate the action of the antibiotic on the whole population: the lever oscillations will stop only when all organisms present on the cantilever are killed by the antibiotic.

Probably the most limiting drawback of the technique is the need to attach the organisms onto the cantilever. It requires chemicals that cross link the organism to the surface of the lever without compromising the cellular physiology. The technique is based on AFM technology and therefore is relatively expensive. However, custom-made devices as depicted in Figure 3 can advantageously replace commercially available AFMs at a fraction of their prices. Particles or bacteria floating in the analysis chamber can cross the laser beam and modify its intensity on the photodiodes. This can lead to a misinterpretation of the phenomena as a displacement of the cantilever [151]. Vibration isolation is an additional issue since AFMs and nanomotion-dedicated devices are relatively sensitive to environmental vibrations. Up to now, MICs and MBCs were only obtained for bactericidal antibiotics and not for bacteriostatic antibiotics. However, we are convinced that a more detailed analysis of the cantilever oscillations should remove this limitation.

## 9. Future Developments

We are confident that nanomotion-based AST will play an important role in specific diagnostic domains such as tuberculosis or septicemia. Currently, several AFM-based nanomotion detectors are implemented for evaluation in the Lausanne University Hospital (Prof. G. Greub, Microbiology Department, CHUV, Switzerland). In the case of positive evaluation, they will be included in the routine diagnosis chain. To further increase nanomotion detection-based AST, potential parallel detection systems should be implemented. This would permit one to probe simultaneously, on a single detector, different antibiotics or different bacterial species. This development would also eliminate the need of any previous knowledge of the bacterial species under evaluation. It would permit one to assess the susceptibility of the microorganism for dozens of different antibiotics simultaneously. A universal cross linker would also greatly facilitate the spread of the device in research medical centers. Finally, additional fundamental knowledge about the origin of the nanomotion is mandatory to permit to the technique to further spread in research centers, pharmaceutical companies and hospitals.

## Figures and Tables

**Table 1:** Examples of current phenotypic antibiotic susceptibility testing (AST) methods that are classified according to the measuring principle: culture-based, optical-based, electrical-based and mechanical-based AST methods. MIC: minimal inhibitory concentration.

Method	Characteristics	Reference
**Culture-based AST methods**		
Broth dilution assay	Macro- or microdilution of medium–antibiotic solution and growth evaluation based on turbidity or colorimetric differences.	[3,4,7,16,17,18,19,20,21,22,23,24,25,26,27]
Disk diffusion	Optical analysis of the resulting colony is based on the growth. MIC determination.	[3,9,10,11]
Gradient diffusion	Similar to the disk diffusion method using a plastic strip.	[38]
Time-kill test	Reveals a time- or concentration-dependent antimicrobial effect drugs synergism or antagonism.	[28,29,30,31,32]
**Optical-based AST methods**		
Optical tracking of cell division	Single-cell division tracking associated with large volume imaging.	[39]
Multiplexed automated digital microscopy	Optical imaging of cells with quantification of growth rates in the presence of antibiotics.	[33,34,35]
oCelloscope	Estimate the growth of bacterial cells with an optical microscope.	[36]
Single-cell morphological analysis (SCMA)	Imaging changes of the morphology of single cells upon antibiotic treatment.	[37]
Surface plasmon resonance (SPR)	A SPR biosensor was used to determine the susceptibility of *Staphylococcus aureus* clinical isolates.	[40]
**Electrical-based AST methods**		
Electric resistance	Growth of cells in a microchannel is directly proportional to the measured resistance change.	[41]
Impedance-based Fast Antimicrobial Susceptibility Test (IFAST)	Changes in biophysical properties of bacteria measured by impedance cytometry.	[42]
Electrochemical	Measurement of the change in current due to electrochemical reactions.	[43,44,45]
Electrical AST (e-AST)	Growth of cells is monitored by detecting capacitance change of bacteria bound to 60 aptamer-functionalized capacitance sensors	[46]
**Mechanical-based AST methods**		
Asynchronous magnetic bead rotation	Detects bacterial growth, based on the rotation of a cluster of magnetic microparticles.	[47]

**Table 2:** Molecular AST methods. SILAC: stable isotope labeling by amino acid in cell culture.

Method	Characteristics	Reference
16S rRNA identification	Influence of antibiotic on growth by measurement of 16S rRNA.	[76]
ATP bioluminescence	ATP quantification as an estimate of the microbial population metabolic activity.	[69,70,71,72,73,74]
DNA microarrays	DNA microarray using 70mer oligonucleotide. probes to detect resistance genes.	[49]
Real-Time PCR	Detection of resistance genes.	[50,51,52,53]
MALDI-TOF MS and broth dilution	Combination of microbial identification with an established AST method.	[65,66]
MALDI-TOF MS and SILAC	Identification of metabolic inactive microorganisms upon antibiotic treatment.	[68]

**Table 3:** Antimicrobial susceptibility testing applications of the AFM cantilever method for pathogenic microorganisms.

Microorganisms	Remark	Antimicrobial	Reference
*Escherichia coli*	Motile bacterium, rapidly growing bacterium	Ampicillin, ceftriaxone, ciprofloxacin	[77,141,142,143]
		Bacteriophage T7	[143]
*Bordetella pertussis*	Non-motile bacterium, slowly growing bacterium	Clarithromycin, ampicillin	[94]
*Staphylococcus aureus*	Non-motile bacterium, rapidly growing bacterium	Ciprofloxacin	[144]
*Mycobacterium abscessus*	Non-motile bacterium, rapidly growing bacterium	Rifampicin, isoniazid, amikacin	[145]
*Bacillus Calmette-Guérin*	Non-motile bacterium, slowly growing bacterium	Rifampicin, isoniazid, amikacin	[145]
*Candida albicans*	Yeast (candidiasis)	Caspofungin	[144,146]

**Table 4:** Other nanomotion detection applications.

Cell/Protein	Remark	Killing/ Neutralizing Agent	Reference
Topoisomerase II	Protein conformational changes are detected	AMPPNP ^1^, aclarubicin	[140]
Mitochondria	Intracellular organelle oscillation detection	Rotenon	[147]
Osteoblasts	Mammalian cell	Glutaraldehyde	[144]
Neurons	Mammalian cell	Osmotic shock	[144]
Breast cancer cells	Mammalian cell	Paclitaxe, doxorubicin	[148,149]
*Arabidopsis thaliana*	Plant cell	Absence of light	[144]

^1^ AMPPNP: adenylyl-imidodiphosphate.

## Data Availability

The data presented in this study are available in Figure 4 and on request from the corresponding author.

## Appendix A

*B. pertussis* Tohama I strain (Collection of Institute Pasteur, Paris, France -CIP 8132-) was grown in Bordetella agar plates with charcoal, supplemented with 7% horse blood, supplied by BD Difco for 48 h at 37 °C. The colonies were cultured for 48 h and then inoculated in 100 mL Erlenmeyers flasks containing 30 mL of Stainer–Sholte (SS) liquid medium. This culture was incubated for 24 h at 37 °C with shaking at 160 rpm. The initial concentration at 650 nm was OD650: 0.2; after 24 h of growth, the culture was centrifuged at 8000 g for 5 min and washed four times with sterile phosphate-buffered saline (PBS). Lastly, bacteria were washed three times in PBS; between each rinse they were sedimented by centrifugation at 8500 rpm for 5 min and were finally suspended in SS liquid medium to get a final high-density bacterial suspension (OD595: 0.5 with 100 µL of suspension diluted in 1 mL of PBS).

Cantilevers of silicon nitride with a force constant of 0.06–0.12 N/m were incubated for 10 min with a drop (10 µL) of 0.5% (*v*/*v*) glutaraldehyde, rinsed with ultrapure water, dried, and incubated with 10 µL of the high-density bacterial suspension. Three washes with PBS were performed to eliminate the non-adhering or poorly adhered bacteria. These cantilevers with adhered bacteria were introduced into the analysis chamber of the nanomotion detector to test different liquid conditions. Before starting the measurements, the analysis chamber was allowed to stabilize for a short time. Initially, the vibration of the cantilever with *B. pertussis* adhered bacteria was analyzed in SS medium and then the liquid was replaced by fresh SS medium with antibiotic and a new data record was carried out, at the room temperature. The antimicrobial used was clarithromycin (Sigma-A3487) in a concentration of 5 µg/mL. The deflection signal was collected at a sampling frequency of 20 kHz (20000 data points by seconds). Cantilever deflection and variance of the deflection signal were calculated to define the amplitude of the sensor’s movements. The evident outliers were removed in cases of inevitable non-biological signal. The information was recorded and analyzed using custom software written in LabVIEW (National Instruments) and the Matlab R2013b software.

## References

[1] WHO (2016). Antimicrobial Resistance: Global Report on Surveillance 2014.

[2] Michael C.A., Dominey-Howes D., Labbate M. (2014). The Antimicrobial Resistance Crisis: Causes, Consequences, and Management. Front. Public Heath.

[3] Jorgensen J.H., Ferraro M.J. (2009). Antimicrobial Susceptibility Testing: A Review of General Principles and Contemporary Practices. Clin. Infect. Dis..

[4] Syal K., Mo M., Yu H., Iriya R., Jing W., Guodong S., Wang S., Grys T.E., Haydel S.E., Tao N. (2017). Current and emerging techniques for antibiotic susceptibility tests. Theranostics.

[5] Van Boeckel T.P., Gandra S., Ashok A., Caudron Q., Grenfell B.T., Levin S.A., Laxminarayan R. (2014). Global antibiotic consumption 2000 to 2010: An analysis of national pharmaceutical sales data. Lancet Infect. Dis..

[6] Van Boeckel T., Laxminarayan R. (2017). Correction to global antibiotic consumption data. Lancet Infect. Dis..

[7] Humphries R.M., Hindler J.A. (2016). Emerging Resistance, new antimicrobial agents … but no tests! the challenge of antimicrobial susceptibility testing in the current us regulatory landscape. Clin. Infect. Dis..

[8] Van Belkum A., Bachmann T.T., Lüdke G., Lisby J.G., Kahlmeter G., Mohess A., Becker K., Hays J.P., Woodford N., Mitsakakis K. (2019). Developmental roadmap for antimicrobial susceptibility testing systems. Nat. Rev. Microbiol..

[9] Horvat R.T. (2010). Review of Antibiogram Preparation and Susceptibility Testing Systems. Hosp. Pharm..

[10] Kronvall G., Giske C.G., Kahlmeter G. (2011). Setting interpretive breakpoints for antimicrobial susceptibility testing using disk diffusion. Int. J. Antimicrob. Agents.

[11] Matuschek E., Brown D.F.J., Kahlmeter G. (2014). Development of the EUCAST disk diffusion antimicrobial susceptibility testing method and its implementation in routine microbiology laboratories. Clin. Microbiol. Infect..

[12] Fournier P.E., Drancourt M., Colson P., Rolain J.M., La Scola B., Raoult D. (2013). Modern clinical microbiology: New challenges and solutions. Nat. Rev. Microbiol..

[13] Dinarelli S., Girasole M., Kasas S., Longo G. (2017). Nanotools and molecular techniques to rapidly identify and fight bacterial infections. J. Microbiol. Methods.

[14] Balouiri M., Sadiki M., Ibnsouda S.K. (2016). Methods for in vitro evaluating antimicrobial activity: A review. J. Pharm. Anal..

[15] Didelot X., Bowden R., Wilson D.J., Peto T.E.A., Crook D.W. (2012). Transforming clinical microbiology with bacterial genome sequencing. Nat. Rev. Genet..

[16] Wiegand I., Hilpert K., Hancock R.E.W. (2008). Agar and broth dilution methods to determine the minimal inhibitory concentration (MIC) of antimicrobial substances. Nat. Protoc..

[17] Lavallée C., Rouleau D., Gaudreau C., Roger M., Tsimiklis C., Locas M.C., Gagnon S., Delorme J., Labbé A.C. (2010). Performance of an agar dilution method and a Vitek 2 card for detection of inducible clindamycin resistance in Staphylococcus spp.. J. Clin. Microbiol..

[18] Gardiner B.J., Grayson M.L., Wood G.M. (2013). Inducible resistance to clindamycin in Staphylococcus aureus: Validation of Vitek-2 against CLSI D-test. Pathology.

[19] Tan Y.E., Ng L.S.Y., Tan T.Y. (2014). Evaluation of Enterococcus faecalis clinical isolates with “penicillin-resistant, ampicillin-susceptible” phenotype as reported by Vitek-2 Compact system. Pathology.

[20] Won E.J., Shin J.H., Kim M.N., Choi M.J., Joo M.Y., Kee S.J., Shin M.G., Suh S.P., Ryang D.W. (2014). Evaluation of the BD Phoenix system for identification of a wide spectrum of clinically important yeast species: A comparison with Vitek 2-YST. Diagn. Microbiol. Infect. Dis..

[21] McGregor A., Schio F., Beaton S., Boulton V., Perman M., Gilbert G. (1995). The microscan walkaway diagnostic microbiology system—An evaluation. Pathology.

[22] Winstanley T., Courvalin P. (2011). Expert systems in clinical microbiology. Clin. Microbiol. Rev..

[23] Snyder J.W., Munier G.K., Johnson C.L. (2008). Direct comparison of the BD phoenix system with the MicroScan WalkAway system for identification and antimicrobial susceptibility testing of Enterobacteriaceae and nonfermentative gram-negative organisms. J. Clin. Microbiol..

[24] Mittman S.A., Huard R.C., Della-Latta P., Whittier S. (2009). Comparison of BD Phoenix to Vitek 2, MicroScan MICroSTREP, and Etest for antimicrobial susceptibility testing of Streptococcus pneumoniae. J. Clin. Microbiol..

[25] Cantón R., Pérez-Vázquez M., Oliver A., Sánchez Del Saz B., Gutiérrez M.O., Martínez-Ferrer M., Baquero F. (2000). Evaluation of the wider system, a new computer-assisted image-processing device for bacterial identification and susceptibility testing. J. Clin. Microbiol..

[26] Swenson J.M., Anderson K.F., Lonsway D.R., Thompson A., McAllister S.K., Limbago B.M., Carey R.B., Tenover F.C., Patel J.B. (2009). Accuracy of commercial and reference susceptibility testing methods for detecting vancomycin-intermediate Staphylococcus aureus. J. Clin. Microbiol..

[27] Junkins A.D., Lockhart S.R., Heilmann K.P., Dohrn C.L., Von Stein D.L., Winokur P.L., Doern G.V., Richter S.S. (2009). BD Phoenix and Vitek 2 detection of mecA-mediated resistance in Staphylococcus aureus with cefoxitin. J. Clin. Microbiol..

[28] Pfaller M.A., Sheehan D.J., Rex J.H. (2004). Determination of Fungicidal Activities against Yeasts and Molds: Lessons Learned from Bactericidal Testing and the Need for Standardization. Clin. Microbiol. Rev..

[29] Konaté K., Mavoungou J.F., Lepengué A.N., Aworet-Samseny R.R.R., Hilou A., Souza A., Dicko M.H., M’Batchi B. (2012). Antibacterial activity against β- lactamase producing Methicillin and Ampicillin-resistants Staphylococcus aureus: Fractional Inhibitory Concentration Index (FICI) determination. Ann. Clin. Microbiol. Antimicrob..

[30] White R.L., Burgess D.S., Manduru M., Bosso J.A. (1996). Comparison of three different in vitro methods of detecting synergy: Time-kill, checkerboard, and E test. Antimicrob. Agents Chemother..

[31] Clancy C.J., Huang H., Cheng S., Derendorf H., Nguyen M.H. (2006). Characterizing the effects of caspofungin on Candida albicans, Candida parapsilosis, and Candida glabrata isolates by simultaneous time-kill and postantifungal-effect experiments. Antimicrob. Agents Chemother..

[32] Klepser M.E., Ernst E.J., Lewis R.E., Ernst M.E., Pfaller M.A. (1998). Influence of test conditions on antifungal time-kill curve results: Proposal for standardized methods. Antimicrob. Agents Chemother..

[33] Chantell C. (2015). Multiplexed Automated Digital Microscopy for Rapid Identification and Antimicrobial Susceptibility Testing of Bacteria and Yeast Directly from Clinical Samples. Clin. Microbiol. Newsl..

[34] Price C.S., Kon S.E., Metzger S. (2014). Rapid antibiotic susceptibility phenotypic characterization of Staphylococcus aureus using automated microscopy of small numbers of cells. J. Microbiol. Methods.

[35] Douglas I.S., Price C.S., Overdier K.H., Wolken R.F., Metzger S.W., Hance K.R., Howson D.C. (2015). Rapid automated microscopy for microbiological surveillance of ventilator-associated pneumonia. Am. J. Respir. Crit. Care Med..

[36] Fredborg M., Andersen K.R., Jørgensen E., Droce A., Olesen T., Jensen B.B., Rosenvinge F.S., Sondergaard T.E. (2013). Real-time optical antimicrobial susceptibility testing. J. Clin. Microbiol..

[37] Choi J., Jung Y.G., Kim J., Kim S., Jung Y., Na H., Kwon S. (2013). Rapid antibiotic susceptibility testing by tracking single cell growth in a microfluidic agarose channel system. Lab Chip.

[38] Brown D.F.J., Brown L. (1991). Evaluation of the e test, a novel method of quantifying antimicrobial activity. J. Antimicrob. Chemother..

[39] Zhang F., Jiang J., McBride M., Yang Y., Mo M., Iriya R., Peterman J., Jing W., Grys T., Haydel S.E. (2020). Direct Antimicrobial Susceptibility Testing on Clinical Urine Samples by Optical Tracking of Single Cell Division Events. Small.

[40] Tawil N., Mouawad F., Lévesque S., Sacher E., Mandeville R., Meunier M. (2013). The differential detection of methicillin-resistant, methicillin-susceptible and borderline oxacillin-resistant Staphylococcus aureus by surface plasmon resonance. Biosens. Bioelectron..

[41] Yang Y., Gupta K., Ekinci K.L. (2020). All-electrical monitoring of bacterial antibiotic susceptibility in a microfluidic device. Proc. Natl. Acad. Sci. USA.

[42] Spencer D.C., Paton T.F., Mulroney K.T., Inglis T.J.J., Sutton J.M., Morgan H. (2020). A fast impedance-based antimicrobial susceptibility test. Nat. Commun..

[43] Ertl P., Robello E., Battaglini F., Mikkelsen S.R. (2000). Rapid antibiotic susceptibility testing via electrochemical measurement of ferricyanide reduction by Escherichia coli and Clostridium sporogenes. Anal. Chem..

[44] Mann T.S., Mikkelsen S.R. (2008). Antibiotic susceptibility testing at a screen-printed carbon electrode array. Anal. Chem..

[45] Onishi K., Enomoto J., Araki T., Takagi R., Suzuki H., Fukuda J. (2018). Electrochemical microdevices for rapid and on-site determination of the minimum inhibitory concentration of antibiotics. Analyst.

[46] Lee K.S., Lee S.M., Oh J., Park I.H., Song J.H., Han M., Yong D., Lim K.J., Shin J.S., Yoo K.H. (2020). Electrical antimicrobial susceptibility testing based on aptamer-functionalized capacitance sensor array for clinical isolates. Sci. Rep..

[47] Kinnunen P., McNaughton B.H., Albertson T., Sinn I., Mofakham S., Elbez R., Newton D.W., Hunt A., Kopelman R. (2012). Self-assembled magnetic bead biosensor for measuring bacterial growth and antimicrobial susceptibility testing. Small.

[48] Frye J.G., Jesse T., Long F., Rondeau G., Porwollik S., McClelland M., Jackson C.R., Englen M., Fedorka-Cray P.J. (2006). DNA microarray detection of antimicrobial resistance genes in diverse bacteria. Int. J. Antimicrob. Agents.

[49] Frye J.G., Lindsey R.L., Rondeau G., Porwollik S., Long F., McClelland M., Jackson C.R., Englen M.D., Meinersmann R.J., Berrang M.E. (2010). Development of a DNA microarray to detect antimicrobial resistance genes identified in the national center for biotechnology information database. Microb. Drug Resist..

[50] Huletsky A., Giroux R., Rossbach V., Gagnon M., Vaillancourt M., Bernier M., Gagnon F., Truchon K., Bastien M., Picard F.J. (2004). New Real-Time PCR Assay for Rapid Detection of Methicillin-Resistant Staphylococcus aureus Directly from Specimens Containing a Mixture of Staphylococci. J. Clin. Microbiol..

[51] Boehme C.C., Nabeta P., Hillemann D., Nicol M.P., Shenai S., Krapp F., Allen J., Tahirli R., Blakemore R., Rustomjee R. (2010). Rapid Molecular Detection of Tuberculosis and Rifampin Resistance. N. Engl. J. Med..

[52] Opota O., Mazza-Stalder J., Greub G., Jaton K. (2019). The rapid molecular test Xpert MTB/RIF ultra: Towards improved tuberculosis diagnosis and rifampicin resistance detection. Clin. Microbiol. Infect..

[53] Chakravorty S., Simmons A.M., Rowneki M., Parmar H., Cao Y., Ryan J., Banada P.P., Deshpande S., Shenai S., Gall A. (2017). The new Xpert MTB/RIF ultra: Improving detection of Mycobacterium tuberculosis and resistance to Rifampin in an assay suitable for point-of-care testing. mBio.

[54] Burckhardt I., Zimmermann S. (2018). Susceptibility Testing of Bacteria Using Maldi-Tof Mass Spectrometry. Front. Microbiol..

[55] Degand N., Carbonnelle E., Dauphin B., Beretti J.L., Le Bourgeois M., Sermet-Gaudelus I., Segonds C., Berche P., Nassif X., Ferroni A. (2008). Matrix-assisted laser desorption ionization-time of flight mass spectrometry for identification of nonfermenting gram-negative bacilli isolated from cystic fibrosis patients. J. Clin. Microbiol..

[56] Seng P., Drancourt M., Gouriet F., La Scola B., Fournier P.E., Rolain J.M., Raoult D. (2009). Ongoing revolution in bacteriology: Routine identification of bacteria by matrix-assisted laser desorption ionization time-of-flight mass spectrometry. Clin. Infect. Dis..

[57] Ledeboer N.A., Hodinka R.L. (2011). Molecular detection of resistance determinants. J. Clin. Microbiol..

[58] Neville S.A., LeCordier A., Ziochos H., Chater M.J., Gosbell I.B., Maley M.W., Van Hal S.J. (2011). Utility of matrix-assisted laser desorption ionization-time of flight mass spectrometry following introduction for routine laboratory bacterial identification. J. Clin. Microbiol..

[59] Dubois D., Leyssene D., Chacornac J.P., Kostrzewa M., Schmit P.O., Talon R., Bonnet R., Delmas J. (2010). Identification of a variety of Staphylococcus species by matrix-assisted laser desorption ionization-time of flight mass spectrometry. J. Clin. Microbiol..

[60] Saleeb P.G., Drake S.K., Murray P.R., Zelazny A.M. (2011). Identification of mycobacteria in solid-culture media by matrix-assisted laser desorption ionization-time of flight mass spectrometry. J. Clin. Microbiol..

[61] Dhiman N., Hall L., Wohlfiel S.L., Buckwalter S.P., Wengenack N.L. (2011). Performance and cost analysis of matrix-assisted laser desorption ionization-time of flight mass spectrometry for routine identification of yeast. J. Clin. Microbiol..

[62] Croxatto A., Prod’hom G., Durussel C., Greub G. (2014). Preparation of a blood culture pellet for rapid bacterial identification and antibiotic susceptibility testing. J. Vis. Exp..

[63] Opota O., Croxatto A., Prod’hom G., Greub G. (2015). Blood culture-based diagnosis of bacteraemia: State of the art. Clin. Microbiol. Infect..

[64] Opota O., Jaton K., Greub G. (2015). Microbial diagnosis of bloodstream infection: Towards molecular diagnosis directly from blood. Clin. Microbiol. Infect..

[65] Kathuria S., Singh P.K., Sharma C., Prakash A., Masih A., Kumar A., Meis J.F., Chowdhary A. (2015). Multidrug-resistant Candida auris misidentified as Candida haemulonii: Characterization by matrix-assisted laser desorption ionization-time of flight mass spectrometry and DNA sequencing and its antifungal susceptibility profile variability by vitek 2, CLSI broth microdilution, and etest method. J. Clin. Microbiol..

[66] Hazelton B., Thomas L.C., Olma T., Kok J., O′Sullivan M., Chen S.C.A., Iredell J.R. (2014). Rapid and accurate direct antibiotic susceptibility testing of blood culture broths using MALDI sepsityper combined with the BD phoenix automated system. J. Med. Microbiol..

[67] Morgenthaler N.G., Kostrzewa M. (2015). Rapid identification of pathogens in positive blood culture of patients with sepsis: Review and meta-analysis of the performance of the Sepsityper kit. Int. J. Microbiol..

[68] Jung J.S., Eberl T., Sparbier K., Lange C., Kostrzewa M., Schubert S., Wieser A. (2014). Rapid detection of antibiotic resistance based on mass spectrometry and stable isotopes. Eur. J. Clin. Microbiol. Infect. Dis..

[69] Finger S., Wiegand C., Buschmann H.J., Hipler U.C. (2013). Antibacterial properties of cyclodextrin-antiseptics-complexes determined by microplate laser nephelometry and ATP bioluminescence assay. Int. J. Pharm..

[70] VOJTEK L., DOBES P., BUYUKGUZEL E., ATOSUO J., HYRSL P. (2014). Bioluminescent assay for evaluating antimicrobial activity in insect haemolymph. Eur. J. Entomol..

[71] Ivančić V., Mastali M., Percy N., Gornbein J., Babbitt J.T., Li Y., Landaw E.M., Bruckner D.A., Churchill B.M., Haake D.A. (2008). Rapid antimicrobial susceptibility determination of uropathogens in clinical urine specimens by use of ATP bioluminescence. J. Clin. Microbiol..

[72] Andreu N., Fletcher T., Krishnan N., Wiles S., Robertson B.D. (2012). Rapid measurement of antituberculosis drug activity in vitro and in macrophages using bioluminescence. J. Antimicrob. Chemother..

[73] Beckers B., Lang H.R.M., Schimke D., Lammers A. (1985). Evaluation of a bioluminescence assay for rapid antimicrobial susceptibility testing of mycobacteria. Eur. J. Clin. Microbiol..

[74] Finger S., Wiegand C., Buschmann H.J., Hipler U.C. (2012). Antimicrobial properties of cyclodextrin-antiseptics-complexes determined by microplate laser nephelometry and ATP bioluminescence assay. Int. J. Pharm..

[75] Galiger C., Brock M., Jouvion G., Savers A., Parlato M., Ibrahim-Granet O. (2013). Assessment of efficacy of antifungals against Aspergillus fumigatus: Value of real-time bioluminescence imaging. Antimicrob. Agents Chemother..

[76] Mach K.E., Mohan R., Baron E.J., Shih M.-C., Gau V., Wong P.K., Liao J.C. (2011). A biosensor platform for rapid antimicrobial susceptibility testing directly from clinical samples. J. Urol..

[77] Longo G., Alonso-Sarduy L., Rio L.M., Bizzini A., Trampuz A., Notz J., Dietler G., Kasas S. (2013). Rapid detection of bacterial resistance to antibiotics using AFM cantilevers as nanomechanical sensors. Nat. Nanotechnol..

[78] Binnig G., Quate C.F., Gerber C. (1986). Atomic force microscope. Phys. Rev. Lett..

[79] Alexander S., Hellemans L., Marti O., Schneir J., Elings V., Hansma P.K., Longmire M., Gurley J. (1989). An atomic-resolution atomic-force microscope implemented using an optical lever. J. Appl. Phys..

[80] Kasas S., Fellay B., Cargnello R. (1994). Observation of the action of penicillin onbacillus subtilis using atomic force microscopy: Technique for the preparation of bacteria. Surf. Interface Anal..

[81] Braga P.C., Ricci D. (1998). Atomic force microscopy: Application to investigation of Escherichia coli morphology before and after exposure to cefodizime. Antimicrob. Agents Chemother..

[82] Braga P.C., Ricci D. (2002). Differences in the susceptibility of Streptococcus pyogenes to rokitamycin and erythromycin A revealed by morphostructural atomic force microscopy. J. Antimicrob. Chemother..

[83] Braga P.C., Ricci D., Dal Sasso M. (2002). Daptomycin morphostructural damage in Bacillus cereus visualized by atomic force microscopy. J. Chemother..

[84] Soon R.L., Nation R.L., Hartley P.G., Larson I., Li J. (2009). Atomic force microscopy investigation of the morphology and topography of colistin-heteroresistant Acinetobacter baumannii strains as a function of growth phase and in response to colistin treatment. Antimicrob. Agents Chemother..

[85] Kim Y.S., Kim K.S., Han I., Kim M.H., Jung M.H., Park H.K. (2012). Quantitative and qualitative analysis of the antifungal activity of allicin alone and in combination with antifungal drugs. PLoS ONE.

[86] Formosa C., Schiavone M., Martin-Yken H., François J.M., Duval R.E., Dague E. (2013). Nanoscale effects of caspofungin against two yeast species, saccharomyces cerevisiae and candida albicans. Antimicrob. Agents Chemother..

[87] Krieg M., Fläschner G., Alsteens D., Gaub B.M., Roos W.H., Wuite G.J.L., Gaub H.E., Gerber C., Dufrêne Y.F., Müller D.J. (2019). Atomic force microscopy-based mechanobiology. Nat. Rev. Phys..

[88] Demir I., Blockx J., Dague E., Guiraud P., Thielemans W., Muylaert K., Formosa-Dague C. (2020). Nanoscale Evidence Unravels Microalgae Flocculation Mechanism Induced by Chitosan. ACS Appl. Bio Mater..

[89] Kumar A., Ting Y.P. (2013). Effect of sub-inhibitory antibacterial stress on bacterial surface properties and biofilm formation. Colloids Surf. B Biointerfaces.

[90] Vadillo-Rodríguez V., Logan B.E. (2006). Localized attraction correlates with bacterial adhesion to glass and metal oxide substrata. Environ. Sci. Technol..

[91] Fang H.H.P., Chan K.Y., Xu L.C. (2000). Quantification of bacterial adhesion forces using atomic force microscopy (AFM). J. Microbiol. Methods.

[92] Dupres V., Menozzi F.D., Locht C., Clare B.H., Abbott N.L., Cuenot S., Bompard C., Raze D., Dufrêne Y.F. (2005). Nanoscale mapping and functional analysis of individual adhesins on living bacteria. Nat. Methods.

[93] Gilbert Y., Deghorain M., Wang L., Xu B., Pollheimer P.D., Gruber H.J., Errington J., Hallet B., Haulot X., Verbelen C. (2007). Single-molecule force spectroscopy and imaging of the vancomycin/D-Ala-D- Ala interaction. Nano Lett..

[94] Villalba M.I., Stupar P., Chomicki W., Bertacchi M., Dietler G., Arnal L., Vela M.E., Yantorno O., Kasas S. (2018). Nanomotion Detection Method for Testing Antibiotic Resistance and Susceptibility of Slow-Growing Bacteria. Small.

[95] Vinckier A., Semenza G. (1998). Measuring elasticity of biological materials by atomic force microscopy. FEBS Lett..

[96] Hertz H. (1882). Ueber die Berührung fester elastischer Körper. J. fur die Reine und Angew. Math..

[97] Sneddon I.N. (1965). The relation between load and penetration in the axisymmetric boussinesq problem for a punch of arbitrary profile. Int. J. Eng. Sci..

[98] Tatara Y. (1989). Extensive theory of force- approach relations of elastic spheres in compression and in impact. J. Eng. Mater. Technol. Trans. ASME.

[99] Cappella B., Dietler G. (1999). Force-distance curves by atomic force microscopy. Surf. Sci. Rep..

[100] Butt H.J., Cappella B., Kappl M. (2005). Force measurements with the atomic force microscope: Technique, interpretation and applications. Surf. Sci. Rep..

[101] Xu W., Mulhern P.J., Blackford B.L., Jericho M.H., Firtel M., Beveridge T.J. (1996). Modeling and measuring the elastic properties of an archaeal surface, the sheath of Methanospirillum hungatei, and the implication for methane production. J. Bacteriol..

[102] Arnoldi M., Kacher C.M., Bäuerlein E., Radmacher M., Fritz M. (1998). Elastic properties of the cell wall of Magnetospirillum gryphiswaldense investigated by atomic forcemicroscopy. Appl. Phys. A Mater. Sci. Process..

[103] Arce F.T., Carlson R., Monds J., Veeh R., Hu F.Z., Stewart P.S., Lal R., Ehrlich G.D., Avci R. (2009). Nanoscale structural and mechanical properties of nontypeable haemophilus influenzae biofilms. J. Bacteriol..

[104] Lau P.C.Y., Dutcher J.R., Beveridge T.J., Lam J.S. (2009). Absolute quantitation of bacterial biofilm adhesion and viscoelasticity by microbead force spectroscopy. Biophys. J..

[105] Wang H., Wilksch J.J., Lithgow T., Strugnell R.A., Gee M.L. (2013). Nanomechanics measurements of live bacteria reveal a mechanism for bacterial cell protection: The polysaccharide capsule in Klebsiella is a responsive polymer hydrogel that adapts to osmotic stress. Soft Matter.

[106] Bailey R.G., Turner R.D., Mullin N., Clarke N., Foster S.J., Hobbs J.K. (2014). The interplay between cell wall mechanical properties and the cell cycle in staphylococcus aureus. Biophys. J..

[107] Arnal L., Serra D.O., Cattelan N., Castez M.F., Vázquez L., Salvarezza R.C., Yantorno O.M., Vela M.E. (2012). Adhesin contribution to nanomechanical properties of the virulent Bordetella pertussis envelope. Langmuir.

[108] Roduit C., Sekatski S., Dietler G., Catsicas S., Lafont F., Kasas S. (2009). Stiffness tomography by atomic force microscopy. Biophys. J..

[109] Roduit C., Saha B., Alonso-Sarduy L., Volterra A., Dietler G., Kasas S. (2012). OpenFovea: Open-source AFM data processing software. Nat. Methods.

[110] Longo G., Rio L.M., Trampuz A., Dietler G., Bizzini A., Kasas S. (2013). Antibiotic-induced modifications of the stiffness of bacterial membranes. J. Microbiol. Methods.

[111] Longo G., Kasas S. (2014). Effects of antibacterial agents and drugs monitored by atomic force microscopy. Wiley Interdiscip. Rev. Nanomed. Nanobiotechnol..

[112] Kasas S., Stupar P., Dietler G. (2018). AFM contribution to unveil pro- and eukaryotic cell mechanical properties. Semin. Cell Dev. Biol..

[113] Garcia R. (2020). Nanomechanical mapping of soft materials with the atomic force microscope: Methods, theory and applications. Chem. Soc. Rev..

[114] Formosa-Dague C., Duval R.E., Dague E. (2018). Cell biology of microbes and pharmacology of antimicrobial drugs explored by Atomic Force Microscopy. Semin. Cell Dev. Biol..

[115] Barnes J.R., Stephenson R.J., Welland M.E., Gerber C., Gimzewski J.K. (1994). Photothermal spectroscopy with femtojoule sensitivity using a micromechanical device. Nature.

[116] Berger R., Gerber C., Gimzewski J.K., Meyer E., Güntherodt H.J. (1996). Thermal analysis using a micromechanical calorimeter. Appl. Phys. Lett..

[117] Boisen A., Dohn S., Keller S.S., Schmid S., Tenje M. (2011). Cantilever-like micromechanical sensors. Rep. Prog. Phys..

[118] Godin M., Tabard-Cossa V., Miyahara Y., Monga T., Williams P.J., Beaulieu L.Y., Bruce Lennox R., Grutter P. (2010). Cantilever-based sensing: The origin of surface stress and optimization strategies. Nanotechnology.

[119] Alvarez M., Lechuga L.M. (2010). Microcantilever-based platforms as biosensing tools. Analyst.

[120] Hansen K.M., Thundat T. (2005). Microcantilever biosensors. Methods.

[121] Waggoner P.S., Craighead H.G. (2007). Micro- and nanomechanical sensors for environmental, chemical, and biological detection. Lab Chip.

[122] Braun T., Ghatkesar M.K., Backmann N., Grange W., Boulanger P., Letellier L., Lang H.-P., Bietsch A., Gerber C., Hegner M. (2009). Quantitative time-resolved measurement of membrane protein–ligand interactions using microcantilever array sensors. Nat. Nanotechnol..

[123] Ilic B., Czaplewski D., Craighead H.G., Neuzil P., Campagnolo C., Batt C. (2000). Mechanical resonant immunospecific biological detector. Appl. Phys. Lett..

[124] Fritz J., Baller M.K., Lang H.P., Rothuizen H., Vettiger P., Meyer E., Güntherodt H.J., Gerber C., Gimzewski J.K. (2000). Translating biomolecular recognition into nanomechanics. Science.

[125] Fritz J. (2008). Cantilever biosensors. Analyst.

[126] Willaert R., Kasas S., Devreese B., Dietler G. (2016). Yeast Nanobiotechnology. Fermentation.

[127] Lang H.P., Baller M.K., Berger R., Gerber C., Gimzewski J.K., Battiston F.M., Fornaro P., Ramseyer J.P., Meyer E., Güntherodt H.J. (1999). An artificial nose based on a micromechanical cantilever array. Anal. Chim. Acta.

[128] Braun T., Barwich V., Ghatkesar M.K., Bredekamp A.H., Gerber C., Hegner M., Lang H.P. (2005). Micromechanical mass sensors for biomolecular detection in a physiological environment. Phys. Rev. E Stat. Nonlinear Soft Matter Phys..

[129] Hosaka S., Chiyoma T., Ikeuchi A., Okano H., Sone H., Izumi T. (2006). Possibility of a femtogram mass biosensor using a self-sensing cantilever. Curr. Appl. Phys..

[130] Godin M., Delgado F.F., Son S., Grover W.H., Bryan A.K., Tzur A., Jorgensen P., Payer K., Grossman A.D., Kirschner M.W. (2010). Using buoyant mass to measure the growth of single cells. Nat. Methods.

[131] Ndieyira J.W., Watari M., Barrera A.D., Zhou D., Vögtli M., Batchelor M., Cooper M.A., Strunz T., Horton M.A., Abell C. (2008). Nanomechanical detection of antibiotic-mucopeptide binding in a model for superbug drug resistance. Nat. Nanotechnol..

[132] Liu Y., Schweizer L.M., Wang W., Reuben R.L., Schweizer M., Shu W. (2013). Label-free and real-time monitoring of yeast cell growth by the bending of polymer microcantilever biosensors. Sens. Actuators B Chem..

[133] Cermak N., Olcum S., Delgado F.F., Wasserman S.C., Payer K.R., Murakami M.A., Knudsen S.M., Kimmerling R.J., Stevens M.M., Kikuchi Y. (2016). High-throughput measurement of single-cell growth rates using serial microfluidic mass sensor arrays. Nat. Biotechnol..

[134] Burg T.P., Godin M., Knudsen S.M., Shen W., Carlson G., Foster J.S., Babcock K., Manalis S.R. (2007). Weighing of biomolecules, single cells and single nanoparticles in fluid. Nature.

[135] Bryan A.K., Goranov A., Amon A., Manalis S.R. (2010). Measurement of mass, density, and volume during the cell cycle of yeast. Proc. Natl. Acad. Sci. USA.

[136] Park K., Jang J., Irimia D., Sturgis J., Lee J., Robinson J.P., Toner M., Bashir R. (2008). “Living cantilever arrays” for characterization of mass of single live cells in fluids. Lab Chip.

[137] Bryan A.K., Hecht V.C., Shen W., Payer K., Grover W.H., Manalis S.R. (2014). Measuring single cell mass, volume, and density with dual suspended microchannel resonators. Lab Chip.

[138] Nugaeva N., Gfeller K.Y., Backmann N., Lang H.P., Düggelin M., Hegner M. (2005). Micromechanical cantilever array sensors for selective fungal immobilization and fast growth detection. Biosens. Bioelectron..

[139] Etayash H., Khan M.F., Kaur K., Thundat T. (2016). Microfluidic cantilever detects bacteria and measures their susceptibility to antibiotics in small confined volumes. Nat. Commun..

[140] Alonso-Sarduy L., De Los Rios P., Benedetti F., Vobornik D., Dietler G., Kasas S., Longo G. (2014). Real-Time Monitoring of Protein Conformational Changes Using a Nano-Mechanical Sensor. PLoS ONE.

[141] Stupar P., Opota O., Longo G., Prod’hom G., Dietler G., Greub G., Kasas S. (2017). Nanomechanical sensor applied to blood culture pellets: A fast approach to determine the antibiotic susceptibility against agents of bloodstream infections. Clin. Microbiol. Infect..

[142] Lissandrello C., Inci F., Francom M., Paul M.R., Demirci U., Ekinci K.L. (2014). Nanomechanical motion of Escherichia coli adhered to a surface. Appl. Phys. Lett..

[143] Mertens J., Cuervo A., Carrascosa J.L. (2019). Nanomechanical detection of: Escherichia coli infection by bacteriophage T7 using cantilever sensors. Nanoscale.

[144] Kasas S., Ruggeri F.S., Benadiba C., Maillard C., Stupar P., Tournu H., Dietler G., Longo G. (2015). Detecting nanoscale vibrations as signature of life. Proc. Natl. Acad. Sci. USA.

[145] Mustazzolu A., Venturelli L., Dinarelli S., Brown K., Floto R.A., Dietler G., Fattorini L., Kasas S., Girasole M., Longo G. (2019). A rapid unraveling of the activity and antibiotic susceptibility of mycobacteria. Antimicrob. Agents Chemother..

[146] Kohler A.-C., Venturelli L., Kannan A., Sanglard D., Dietler G., Willaert R., Kasas S. (2020). Yeast Nanometric Scale Oscillations Highlights Fibronectin Induced Changes in C. albicans. Fermentation.

[147] Stupar P., Chomicki W., Maillard C., Mikeladze D., Kalauzi A., Radotić K., Dietler G., Kasas S. (2017). Mitochondrial activity detected by cantilever based sensor. Mech. Sci..

[148] Wu S., Liu X., Zhou X., Liang X.M., Gao D., Liu H., Zhao G., Zhang Q., Wu X. (2016). Quantification of cell viability and rapid screening anti-cancer drug utilizing nanomechanical fluctuation. Biosens. Bioelectron..

[149] Kasas S., Stupar P., Longo G., Dietler G. (2015). Détecter la vie grâce à la microscopie à force atomique. Médecine/Sciences.

[150] Venturelli L., Kohler A.C., Stupar P., Villalba M.I., Kalauzi A., Radotic K., Bertacchi M., Dinarelli S., Girasole M., Pešić M. (2020). A perspective view on the nanomotion detection of living organisms and its features. J. Mol. Recognit..

[151] Bennett I., Pyne A.L.B., McKendry R.A. (2020). Cantilever Sensors for Rapid Optical Antimicrobial Sensitivity Testing. ACS Sens..

